# Large Head in Asymptomatic Child: A Subtle Presentation of Connective Tissue Disorder With Spontaneous Significant Intracerebral Bleed

**DOI:** 10.7759/cureus.29192

**Published:** 2022-09-15

**Authors:** Badriah G Alasmari, Muhammad Saeed, Mohammad H Alsumaili, Ali M Tahir

**Affiliations:** 1 Department of Paediatrics, Armed Forces Hospital Southern Region, Khamis Mushait, SAU

**Keywords:** asymptomatic, marfan syndrome, connective tissue disorders, subdural hematoma, large head size

## Abstract

Three years old boy with reassuring development had presented to the Pediatric Neurology clinic with a referral due to a large head. Occipito-frontal circumference was more than 97^th^ centile with an unremarkable neurological examination. MRI brain exhibited an acute on chronic large right frontoparietal subdural hematoma with prominent mass effect. Consequentially, the hematoma was evacuated by the neurosurgeon. Postoperative recovery stayed satisfactory. Hematology workup showed normal coagulation and clotting factors levels. Whole exome sequencing (WES) study revealed heterozygous variant c.5187G>A p.(Trp1729*) in gene *FBN1* - pathogenic for Marfan syndrome. However, this variant has not yet been reported in association with cerebral arteritis/intracerebral bleed. On follow-up, the child remained asymptomatic clinically with static head size. This drags us towards the fact that significant yet asymptomatic spontaneous intracerebral hemorrhage can be an infrequent presentation in pediatrics in regard to connective tissue disorders. Moreover, children with Marfan syndrome having variant c.5187G>A p.(Trp1729*) of gene *FBN1 *can have a rare presentation with cerebral arteritis or intracerebral bleed.

## Introduction

Marfan syndrome is a rare connective tissue disorder resulting from a defect in the FBN1 gene. The reported prevalence is 1:5000 to 1:10000. Clinically, it is characterized by the involvement of cardiovascular, musculoskeletal, ophthalmic, or pulmonary systems [1].

Large head size entails as a common entity in Pediatrics. It is defined as occipitofrontal circumference (OFC) of more than 99.6th centile for age and gender [2]. Usually, the associated developmental disorder or raised intracranial pressure (ICP) signs are pressing considerations for urgent investigations [3]. Here we are presenting a case of Marfan syndrome, who presented with the isolated feature of large head size without any associated clinical features.

## Case presentation

We present a case of three years old boy of Arab ethnicity, who presented to the Pediatric Neurology Clinic as a referral from General Pediatrics due to parental concern for his large head size. Parents noticed a progressive increase in head size since six months of age. The child was delivered at term with no admission to the Neonatal Intensive Care Unit (NICU). The child's development was appropriate for his age, with no regression of milestones or any behavioral change noticed recently. There was no history of any convulsion, headache, irritability, vomiting, or any significant head trauma, and without any hospital admission in the past. There was no family history of any genetic syndromes or developmental problems/learning disabilities. The OFC was found to be more than 97^th^ centile for his age (57 cm), while the weight of the child was recorded at 50^th^ centile for his age and gender. The detailed neurological assessment showed normal motor and sensory components, with a normal gait, spine, and coordination, and a lack of neurocutaneous stigmata with no dysmorphism. The systemic review was unremarkable, with the absence of visceromegaly. Fundoscopy ruled out any papilloedema.

**Figure 1:**
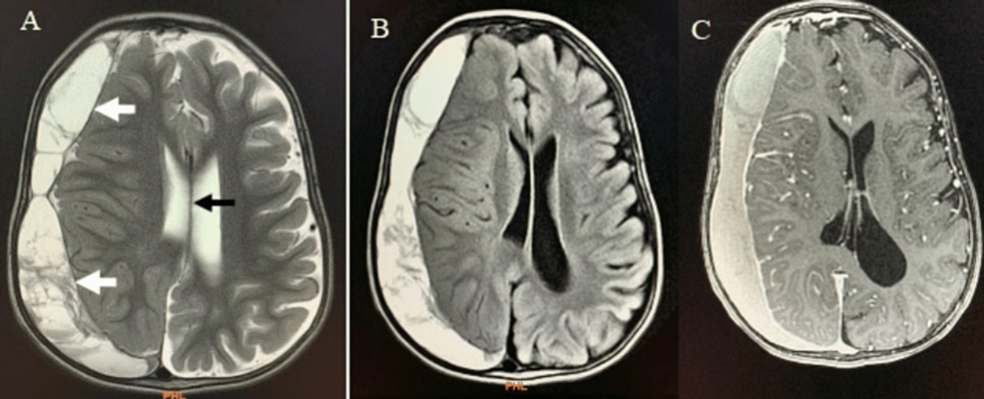
(A) Axial section of brain MRI T2 showing large subdural hematoma (white arrows) in right-sided frontal and temporal lobes with marked positive mass effect and midline shift (black arrow) (B) Axial section of brain MRI FLAIR and (C) Axial section of brain MRI contrast-enhanced.

The inquisitive sense of the neurologist dragged him towards certain definite investigations. MRI brain was requested for any dilated ventricles/hydrocephalus. MRI brain exhibited an acute on chronic large right frontoparietal subdural hematoma (1.8 x 10.9 x 6.4 cm) with prominent mass effect on surrounding structures with effacement of underlying cortical sulci and midline shift of 1.2 cm (Figure 1).

Hematology workup revealed normal coagulation, clotting factors levels, and thyroid functions.

**Figure 2:**
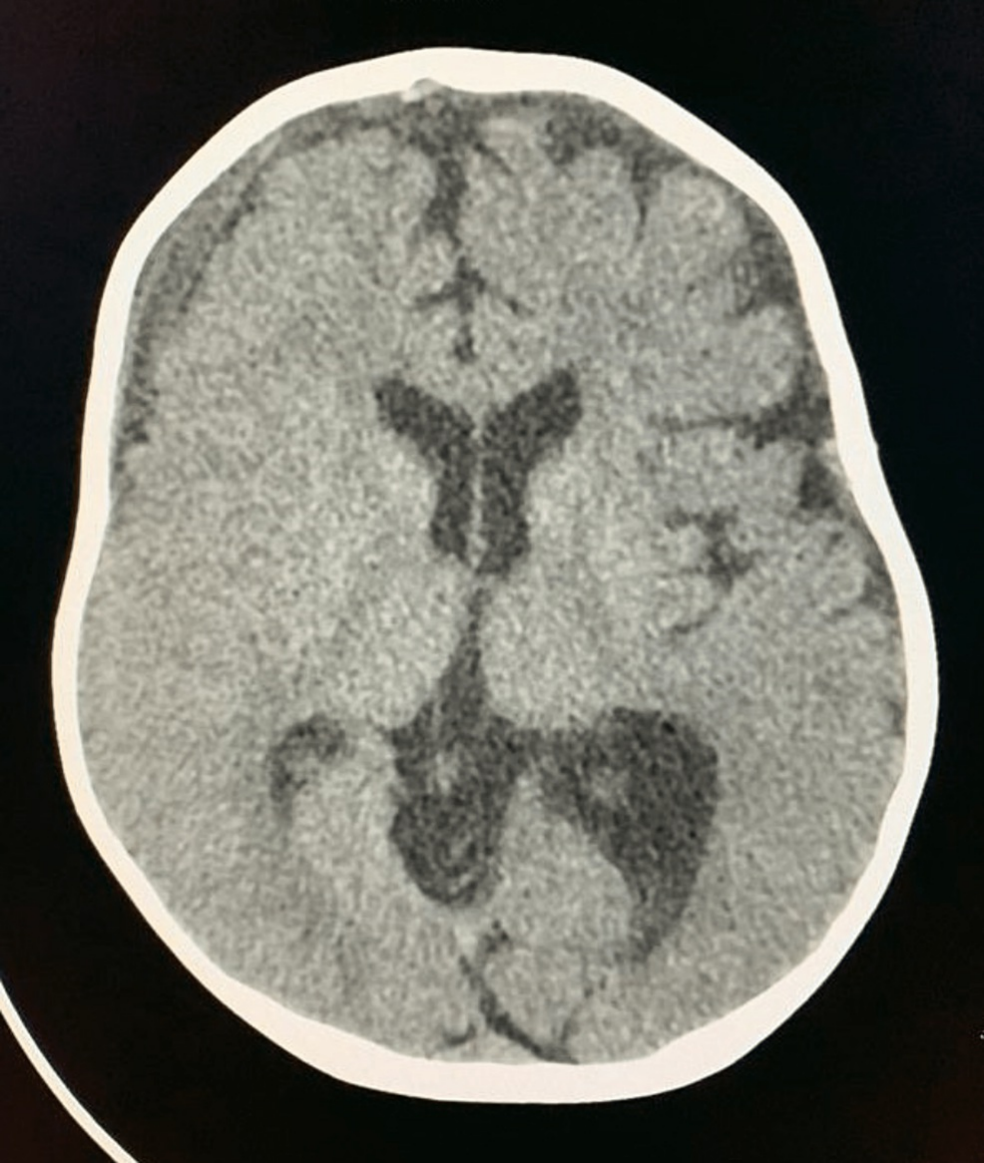
Axial section of brain CT (post evacuation of subdural hematoma) with no mass effect.

The neurosurgeon was approached and intervened with craniotomy for evacuation of right-sided subdural hematoma. The postoperative recovery of the child remained quite uneventful. The drain was removed on second postoperative day, and follow-up scan (CT brain) showed no further collection and without any mass effect (Figure 2).

The child was discharged with no complications or any neurological deficit. Whole Exome Sequencing (WES) study identified the heterozygous variant c.5187G>A p.(Trp1729*) in the FBN1 gene - pathogenic for autosomal dominant Marfan syndrome. During the outdoor follow-up visit, the child was found to be asymptomatic and with no neurological deficit.

## Discussion

Incidental findings on neuroimaging carry a significant burden of uncalled for investigations/interventions. However, in certain cases, such findings result in timely intervention before resulting in debilitating sequelae [4]. We witnessed a significant intracerebral bleed with midline shift in this asymptomatic and developmentally normal child. This infers to the fact that inquisitive acumen of an experienced physician can still herald towards further considerations on priority in selected individuals. Biswas et al. have mentioned intracerebral hemorrhage happening in child clinically quite normal [5]. 

In our pursuit of identifying the cause, we identified the FBN1 gene as pathogenic for Marfan syndrome. Abbas et al., while evaluating intracerebral hemorrhage in children, identified hematologic disorder in 52%, vascular malformation in 14%, and idiopathic in 26%, while neurosurgery was undertaken in 41% of cases [6]. During consideration of connective tissue disorder as the cause, Marfan Syndrome can involve a wide range of organs with varying symptoms. It encompasses the potential for varied complications entailing the heart, aorta, skeletal system, eyes, spinal cord, and brain. The neurovascular complications witnessed in Marfan Syndrome include TIA (65%), and ischemic stroke (10%), while subdural hematoma in 10% of cases [7].

As reported by Kodolitsch et al., while considering the cerebral arteritis in Marfan Syndrome, the pathogenic FBN1 variant has not been mentioned in the literature. This novel variant c.5187G>A p.(Trp1729*) of gene FBN1 can serve in identifying patients with connective tissue disorder with the potential for spontaneous intracerebral hemorrhage [8].

## Conclusions

In conclusion, large-sized head in children needs to be thoroughly evaluated, even in cases with normal development and reassuring detailed examination. Moreover, significant spontaneous intracerebral hemorrhage can be identified in clinically asymptomatic patients. In this case, we identified a novel variant c.5187G>A p.(Trp1729*) in gene FBN1, which was eventually linked to intracranial hemorrhage in connective tissue disorder (Marfan syndrome). This may expand the genetic horizon at one end while assisting in the timely identification of at-risk children by the sensitized primary physician. This will also help in the pursuit of highly desired genetic counseling for patients and families.
